# Development and Validation of a Novel Risk Prediction Model Using Recursive Feature Elimination Algorithm for Acute-on-Chronic Liver Failure in Chronic Hepatitis B Patients With Severe Acute Exacerbation

**DOI:** 10.3389/fmed.2021.748915

**Published:** 2021-11-01

**Authors:** Mingxue Yu, Xiangyong Li, Yaxin Lu, Yusheng Jie, Xinhua Li, Xietong Shi, Shaolong Zhong, Yuankai Wu, Wenli Xu, Zifeng Liu, Yutian Chong

**Affiliations:** ^1^TheDepartment of Infectious Disease, The Third Affiliated Hospital of Sun Yat-sen University, Guangzhou, China; ^2^The Department of Clinical Data Center, The Third Affiliated Hospital of Sun Yat-sen University, Guangzhou, China; ^3^The Department of Infectious Disease, Jieyang People's Hospital (Jieyang Affiliated Hospital of Sun Yat-sen University), Jieyang, China

**Keywords:** prediction model, machine learning, recursive feature elimination algorithm, chronic hepatitis B, acute exacerbation, acute-on-chronic liver failure (ACLF)

## Abstract

**Background:** Patients with chronic hepatitis B (CHB) with severe acute exacerbation (SAE) are at a progression stage of acute-on-chronic liver failure (ACLF) but uniform models for predicting ACLF occurrence are lacking. We aimed to present a risk prediction model to early identify the patients at a high risk of ACLF and predict the survival of the patient.

**Methods:** We selected the best variable combination using a novel recursive feature elimination algorithm to develop and validate a classification regression model and also an online application on a cloud server from the training cohort with a total of 342 patients with CHB with SAE and two external cohorts with a sample size of 96 and 65 patients, respectively.

**Findings:** An excellent prediction model called the PATA model including four predictors, prothrombin time (PT), age, total bilirubin (Tbil), and alanine aminotransferase (ALT) could achieve an area under the receiver operating characteristic curve (AUC) of 0.959 (95% CI 0.941–0.977) in the development set, and AUC of 0.932 (95% CI 0.876–0.987) and 0.905 (95% CI 0.826–0.984) in the two external validation cohorts, respectively. The calibration curve for risk prediction probability of ACLF showed optimal agreement between prediction by PATA model and actual observation. After predictive stratification into different risk groups, the C-index of predictive 90-days mortality was 0.720 (0.675–0.765) for the PATA model, 0.549 (0.506–0.592) for the end-stage liver disease score model, and 0.648 (0.581–0.715) for Child–Turcotte–Pugh scoring system.

**Interpretation:** The highlypredictive risk model and easy-to-use online application can accurately predict the risk of ACLF with a poor prognosis. They may facilitate risk communication and guidetherapeutic options.

## Introduction

Chronichepatitis B virus (HBV) infection poses a global health challenge (1). Hepatitis activity with alanine aminotransferase (ALT) elevation, also called acute exacerbation or hepatitis flare, may occur spontaneously either over the natural course of the disease or following therapy among chronic HBV infection (2, 3). Up to 30% of patients with chronic hepatitis B (CHB) experience hepatitis reactivation every year (4), and some patients will experience severe acute exacerbation (SAE), accompanied by jaundice and hepatic decompensation (5–7). Indeed, compelling evidence shows that SAE has been proposed following the prewarning signs of HBV-related acute-on-chronic liver failure (ACLF) and has been considered a progressive stage in the development of ACLF (5–9). ACLF is a common acute deterioration of hepatic function syndrome, and the short-term in-hospital mortality rate is over 70% if emergency liver transplantation is not available (9). Although liver transplantation is the only effective treatment for ACLF, due to the high cost and shortage of liver source, only a small number of patients undergo liver transplantation (10). In this situation, it is believed that early identification of the high risk of ACLF is of vital importance so that physicians can focus and intervene in advance to slow down or stop the progression of SAE to ACLF (9, 11, 12) and improve the prognosis of the patient.

However, uniform criteria for predicting ACLF occurrence are lacking and patients who are truly at the risk of ACLF are still ill-defined. There are currently several models to evaluate the severity and prognosis of patients with severe liver disease, including the Child–Turcotte–Pugh (CTP) scoring system, the model for end-stage liver disease (MELD), the sequential organ failure assessment score (SOFA), and other predictive models, but none of them have been universally accepted for predict accurate incidence of ACLF (13–15). First, majority scoring systems were originally applied for the evaluation of liver disease severity to predict the outcome of patients. Second, most of these existing models were established among European and American populations. The etiology of ACLF varies with geographic location (16). The leading cause of ACLF among European and American patients is alcohol consumption, whereas among Asian patients is the infection of HBV (12, 16). Risk equations and risk functions are widely applied in patient management, clinical diagnosis, risk stratification, treatment selection, and prognosis prediction (17, 18). However, for new mathematical prediction models for ACLF, there is no model for external multicenter validation. These questions reflect high-priority areas for an accurate prediction model of HBV-ACLF.

Recursive feature elimination (RFE) algorithm (19) is an innovative machine learning algorithm and a backward selection procedure to determine if predictors would be advantageous and select the best predictors (according to the coefficient) to establish the model. To date, this method had not been used in the risk assessment of ACLF patients. In this study, in order to help physicians early identify high-risk patients with CHB of HBV-ACLF, we developed and validated a simple model in three independent cohorts by utilizing RFE analysis. Furthermore, for assessing the prognosis of patients, we compared the performance of the model in predicting 90-days mortality with MELD score and CTP score. The results of this study may further guide and optimize therapeutic strategies for SAE patients with CHB. To the best of our knowledge, our study is the first report of a polycentric risk prediction model in patients with CHB with SAE.

## Materials and Methods

The methods described in this article are in accordance with the Transparent reporting of a multivariable prediction model for individual prognosis or diagnosis (TRIPOD) statement (20).

### Study Design and Population

**Figure 1:**
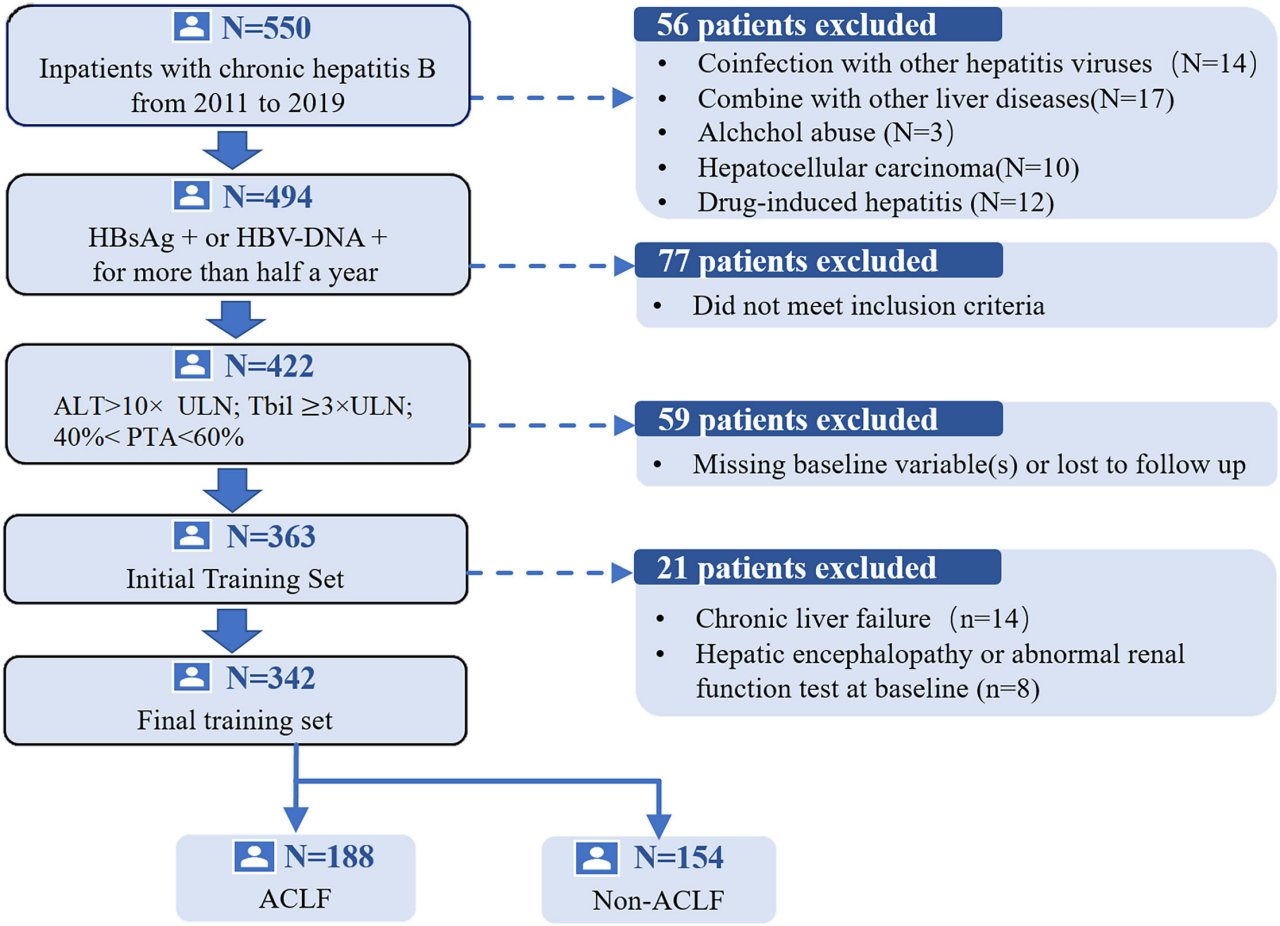
The flow chart of the study group selection process.HBsAg, hepatitis B surface antigen; ALT, alanine aminotransferase; ACLF, acute-on-chronic liver failure; PTA, prothrombin activity; TBil, total bilirubin; ULN, upper limit of normal.

We performed a multicenter retrospective cohort study. Data were collected in three independent hospitals. A total of 342 SAE patients with CHB were enrolled in the study as the development cohort from the Third Affiliated Hospital of Sun Yat-sen University in Guangzhou, China between 2011 and 2019. The preliminary screening identified 550 CHB hospitalized patients with ALT levels elevated. Patients who did not meet the research standards were excluded (*n* = 208). The flow chart of the training group selection process is presented in Figure 1. Patients in the validation cohorts were from two different geographic hospitals, namely Yuedong Hospital of the Third Affiliated Hospital of Sun Yat-sen University in Meizhou, China, between 2016 and 2020 and Jieyang People's Hospital in Jieyang, China, between 2014 and 2019, with a sample size of 96 and 65 patients, respectively, using the same inclusion and exclusion criteria as the development cohort.

### Ethics Statement

This study was approved by the Ethics Committee of the Third Affiliated Hospital of Sun Yat-sen University [(2018)02-384-01]. This study was conducted according to the Declaration of Helsinki. All adult participants provided written informed consent.

### Diagnostic Criteria

The inclusion criteria for SAE of CHB in both the development cohort and validation cohorts were based on those proposed by Tsubota et al. (21) and Wong et al. (22). The inclusion criteria were as follows: (1) presence of hepatitis B surface antigen and HBV DNA for >6 months before hospitalization; (2) ALT >10 × the upper limit of normal (ULN); (3) total bilirubin (Tbil) ≥3 × ULN; (4) 40% < prothrombin time activity (PTA) <60%. The exclusion criteria were coinfections with hepatitis A, C, D, or E viruses, or other viruses including HIV, cytomegalovirus, and Epstein-Barr virus; coexistence of other liver diseases, such as autoimmune liver disease, alcoholic hepatitis, drug-induced liver injury (DILI), and non-alcoholic steatohepatitis (NASH); concurrent diseases resulting in bilirubin elevation, such as hemolytic jaundice, non-hemolytic jaundice, and obstructive jaundice; metabolic liver diseases, including Wilson's disease and hemochromatosis; malignant tumors; and serious extrahepatic diseases. Those patients who had hepatic encephalopathy, ascites, abnormal renal function test at baseline, or did not have follow-up data were also excluded.

Acute-on-chronic liver failure was defined as jaundice (serum bilirubin >5 mg/dL) and coagulopathy (INR >1.5 or prothrombin activity <40%), complicated with ascites and/or encephalopathy as determined by a physical examination in 4 weeks based on the consensus recommendations of the Asian Pacific Association for the study of the liver (APASL 2019) (23). The MELD score was calculated according to the following formula: MELD score = 3.78 × ln[TBil (mg/dL)] +11.2 × ln [INR]+9.57 × ln [Cr(mg/dL)] + 6.43 × (constant for liver disease etiology = 0 if cholestatic or alcoholic, otherwise = 1). The modified CTP score included five parameters: TBil level, albumin level, PT, and the presence and severity of ascites and encephalopathy (24). Liver cirrhosis was defined as coarse liver echotexture with nodularity and small liver size and the presence of features of portal hypertension (e.g., ascites, splenomegaly, and varices) by ultrasound (22).

### Patient Management

All patients were managed by the attending physicians according to international and local guidelines and received conservative therapy. The therapy included bed rest, antiviral therapy, liver-protective treatment, nutritional and energy supplements, intravenous plasma and albumin infusions, water-electrolyte and acid-base equilibrium maintenance, and the prevention and treatment of complications.

Patients in the development cohort were all monitored regularly and followed until death, liver transplantation, or at least for 90 days. The patients who received liver transplantation within 90 days were considered dead and more than 90 days as survival (25, 26). Unfortunately, survival records in validation cohorts were not obtained.

### Predictors

Predictors were collected using an online electronic case report form, and their integrity was systematically checked before being entered into the model. We selected the predictors from the electronic health records based on published literature (27–29) and our clinical experience. Baseline data were the data obtained at the first diagnosis of SAE of CHB from the computerized and paper medical records. We collected the data from patients with complete clinical, laboratory, and follow-up data. Data collection included demographics, basic diseases, precipitating factors, viral tests, liver function, etc. Laboratory variables included aspartate transaminase (AST), ALT, Tbil, albumin (ALB), prothrombin time (PT), fibrinogen (Fib), white blood cell count (WBC), hemoglobin (HGB), platelet (PLT), creatinine (Cr), quantitative determination of hepatitis Bsantigen (HBsAg), hepatitis B e antigen (HBeAg), and HBV DNA levels.

### Data Preprocessing

Missing data were imputed with the mean of continuous variables and the mode of categorical variables. Missing data fill rates in the development set and the two external validation queues are 7, 6, and 5%, respectively (Supplementary Figure 1). Prior to modeling, the Yeo-Johnson (30) transformation was first applied to the raw data, followed by centralization and normalization (Supplementary Table 1).

### Variable Selection

To explore the predictive power of individual variables, we first developed a univariate logistic model for each variable. Since the receiver operator characteristic (ROC) curves are not affected by monotonic transformations of predictors, the area under the receiver operating characteristic curve (AUC) is used as the measure of the strength of the association between predictors and outcomes. A better model was indicated with a higher AUC value, and a perfect model was indicated with an AUC value of 1. The AUC for each model was compared with the null model. Each variable with a *p-*value below 0.05 in the univariate analysis was entered into the model. Next, a quadratic term was applied to continuous variables to evaluate the non-linearity assumption. Subsequently, we used RFE algorithms described in Supplementary Methods for model variable and interaction selection (19). To reduce the risk of overfitting, a resampling algorithm with five repeats of 10-fold cross-validation were performed.

### Model Development, Evaluation, and External Validation

We developed a logistic regression model for the optimal combination of variables ultimately selected by the RFE algorithm and evaluated model performance using AUC, precision-recall (PR) curves, sensitivity, specificity, accuracy, positive predictive values (PPV), negative predictive values (NPV), and brier scores (31). Hosmer-Lemeshow (32) tests were used to assess goodness-of-fit. We plotted the calibration curves by calculating the predicted and true probabilities. The closer the calibration curve is to the 45° diagonal, the better the model performs. We selected a threshold for the balance of sensitivity and specificity on the development set and used this threshold for the geographical external validation at two different hospitals. In addition, the methodology of the model updating to external validation is exhibited in Supplementary Methods.

### Online Application

Nomograms are a graphical representation of predictive statistical models for individual patients (33). Nomogram scoring system based on the results of optimal combination using the RMS package in R version 3.6.2 (34) and web page calculator by using package Shiny for R statistical software (35) were created. Then, we developed an online app to facilitate the use of the data and results from the study. It consists of a website interface to make the results flexible and easily accessible. This application can be accessed and used by physicians.

### Statistical Analysis

For continuous variables, data were described as mean (SD) or median (interquartile spacing), whereas categorical variables were presented as frequencies. Data preprocessing were constructed by R package recipes (36). RFE algorithm was performed using the RFE function in the caret (37) package. The brier score (38) was calculated using the Brier score function in the DescTools (39). We used the survminer (40) package to draw the Kaplan–Meier survival curves (41), calculated the log-rank *p*-value, and used the concordance index (C-index) (41), and also 95% CI as the evaluation index of comparing survival probability. All statistical analyses were performed using the R version 3.6.2 software (Institute for Statistics and Mathematics, Vienna, Austria; http://www.r-project.org) (42). All results were considered statistically significant at *P* < 0.05.

## Results

### Characteristics of Patients

The clinicopathological baseline characteristics of the patients in each cohort are listed in Table 1. A total of 503 patients were enrolled in the study, including 446 (88.7%) men and 57 (11.3%) women. There were 196 (39.0%) patients with HBeAg positive and 130 (38.0%), 25 (26.0%), 12 (18.5%) patients had liver cirrhosis in the development cohort and two validation cohorts, respectively. In the development cohorts, precipitating event of 242 (67.2%) cases was a spontaneous hepatitis B flare-up. The other precipitating events of HBV reactivation were infection in 19.5% of the patients, inappropriate withdrawal of nucleos(t)ide analogs in 5.2% of cases, history of alcohol intake before hospitalization in 5.8% of cases, and the use of hepatotoxic herbal medications in 2.3% of cases. There were 482 patients who received antiviral therapy including entecavir, lamivudine, or telbivudine within 3 days of admission according to their HBV replication levels and willingness, while 21 patients refused to receive antiviral therapy, and 12 patients received artificial liver support system therapy. The mean number of days between hospital admission and the development of ACLF was 7.9 days (range, 2–28days). We observed 188 (55%), 33 (34.4%), 13 (20%) ACLF events within 4 weeks in the development cohort and two in the validation cohorts, respectively. The mean follow-up time was 118.1 weeks (range 12–196 weeks) for the primary cohort. The survival rate of all patients at 90 days was 292 (85.38%). There were 16 patients in the training cohort who received liver transplantation, and 34 patients died within 3 months.

**Table 1:** Clinicalcharacteristics of study participants.

**Characteristics**	**Development Cohort**		**Validation Cohort 1**		**Validation Cohort 2**	
	**(*n = 342)***		**(*n = 96*)**		**(*n = 65)***	
	**Liver failure**	**Non-liver failure**	**Liver failure**	**Non-liver failure**	**Liver failure**	**Non-liver failure**
No. of Patient, *N* (%)	188 (55.0%)	154 (45.0%)	33 (34.4%)	63 (65.6%)	13 (20.0%)	52 (80.0%)
Age (year), median [Q1, Q3]	46.0 [38.0–53.0]	38.0 [33.0–45.8]	45.0 [37.0–56.0]	39.0 [33.0–48.0]	58.0 [51.0–65.0]	37.5 [27.8–49.0]
Gender, N (%)						
Male	170 (90.4%)	139 (90.3%)	28 (84.8%)	53 (84.1%)	13 (100%)	43 (82.7%)
Female	18 (9.57%)	15 (9.74%)	5 (15.2%)	10 (15.9%)	0 (0.00%)	9 (17.3%)
BMI, median [Q1, Q3]	23.1 [21.5–25.4]	22.2 [20.0–24.8]	25.2 [21.7–27.6]	23.1 [20.0–24.7]	–	–
ALB (g/L), median [Q1, Q3]	34.1 [30.6–36.9]	38.3 [34.5–40.8]	34.7 [30.5–37.6]	38.5 [35.3–42.0]	32.0 [30.3–37.6]	38.7 [35.9–41.0]
ALT (U/L), median [Q1, Q3]	688 [251–1440]	874 [483–1560]	1,401 [997–2268]	1,223 [969–1,714]	1,181 [847–1,517]	1,340 [997–1,886]
AST (U/L), median [Q1, Q3]	496 [164–1,004]	511 [258–929]	569 [343–1,110]	497 [285–858]	859 [591–1,398]	706 [473–1,333]
PT (s), median [Q1, Q3]	21.4 [19.4–27.1]	15.5 [14.4–17.4]	20.4 [18.0–26.7]	13.2 [11.9–14.9]	19.0 [17.8–19.6]	14.2 [12.6–16.5]
TB (μmol/L), median [Q1, Q3]	304 [215–388]	106 [49.8–180]	139 [79.9–235]	67.8 [38.1–154]	237 [154–348]	100 [51.9–195]
Fibrinogen (g/L), median [Q1, Q3]	1.79 [1.48–2.24]	2.26 [1.98–2.61]	1.56 [1.27–1.85]	1.98 [1.62–2.38]	1.54 [1.40–1.69]	1.92 [1.58–2.23]
HGB (g/L), median [Q1, Q3]	130 [114–142]	141 [128–151]	130 [123–151]	141 [134–152]	143 [121–150]	140 [126–150]
PLT (10^9^/L), median [Q1, Q3]	124 [95.0–168]	170 [138–211]	136 [124–177]	174 [144–208]	135 [89.0–189]	140 [122–186]
WBC (10^9^/L), median [Q1, Q3]	6.94 [5.56–9.18]	6.06 [4.84–7.51]	6.72 [5.72–9.00]	6.00 [5.28–8.62]	6.87 [5.97–9.81]	6.17 [5.38–8.52]
Cr (μmol/L), median [Q1, Q3]	70.0 [62.2–81.0]	73.0 [66.0–81.6]	61.8 [56.4–75.6]	66.7 [58.9–75.0]	68.0 [52.0–79.0]	66.0 [60.8–73.5]
HBsAg (IU/mL), median [Q1, Q3]	7.65 [5.76–9.06]	8.35 [7.16–8.99]	5.99 [5.52–5.99]	5.99 [5.58–5.99]	7.82 [7.82–7.82]	7.82 [6.42–7.82]
HBeAg positive, N (%)	59 (31.4%)	84 (54.5%)	10 (30.3%)	25 (39.7%)	4 (30.8%)	14 (26.9%)
HBeAg negative, N (%)	129 (68.6%)	70(45.5%)	23(69.7%)	38 (60.3%)	9 (69.2%)	38 (73.1%)
Log (HBV-DNA), median [Q1, Q3]	13.4 [9.94–17.0]	16.0 [12.9–17.7]	12.4 [8.77–17.2]	14.5 [11.2–16.3]	12.1 [11.9–12.3]	13.4 [10.2–16.4]

### Variable Selection

Univariate analysis showed that AST, creatinine, and Gender with *p* > 0.05 were eliminated (Supplementary Table 2). There was no evidence for non-linear relationships for any continuous predictors and no significant interaction effects for the model. To ensure the stability of the model, we also removed Fib that had the absolute correlation coefficient value >0.6 with PT. Finally, according to the results of the RFE algorithm, we selected the best combination from the remaining 12 candidate variables which were PT, age, TBil, and ALT (Supplementary Table 3 and Supplementary Figure 3).

### Model Performance and Validation for Predicting ACLF Development

**Figure 2:**
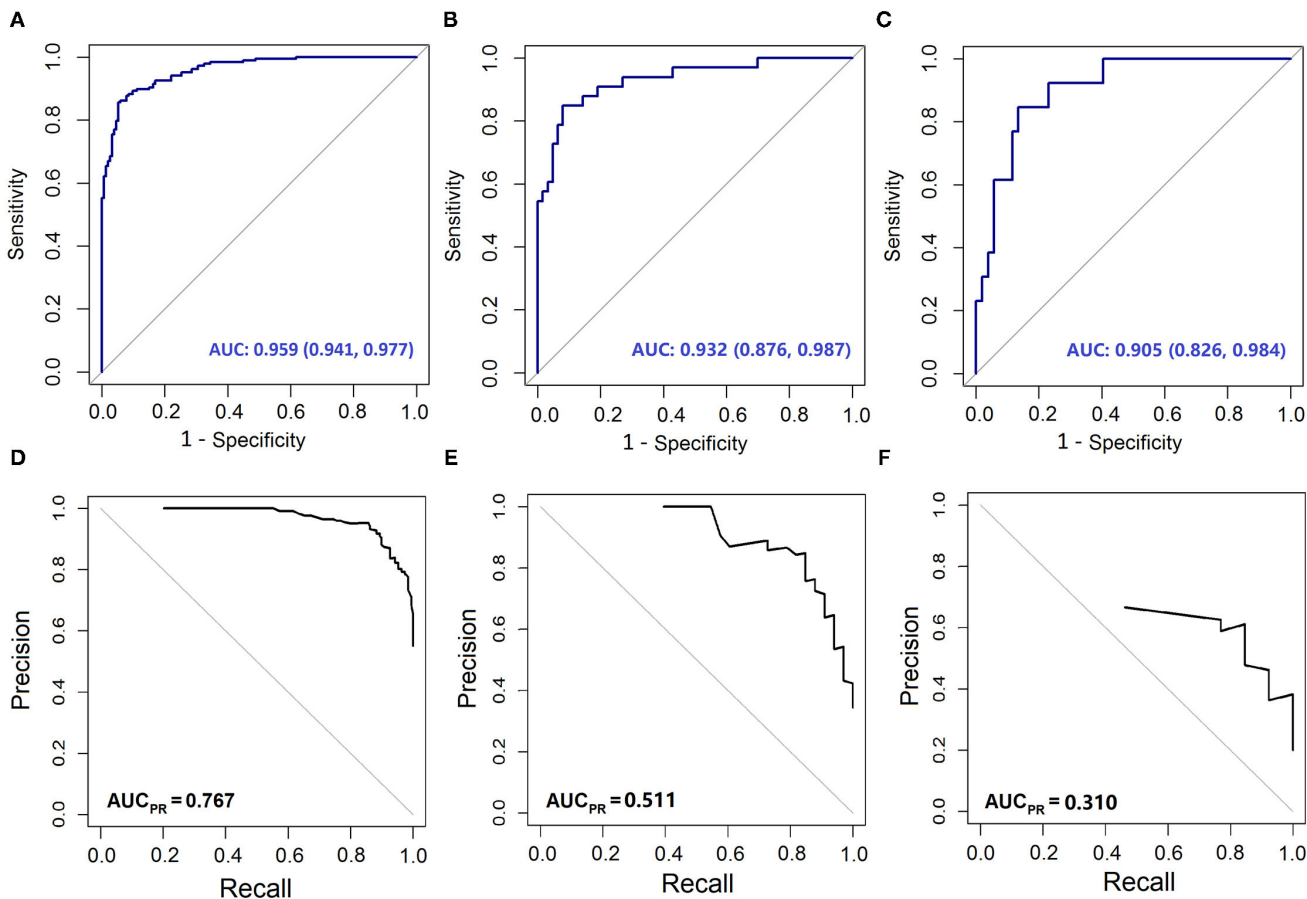
The Area Under the receiver operating characteristic Curve (AUC) and Precision-Recall (PR) curves of different models. The AUC curves of **(A)** development set; **(B)** validation cohort 1; **(C)** validation cohort 2. The PR curve of **(D)** development set; **(E)** validation cohort 1; **(F)** validation cohort 2.

**Figure 3:**
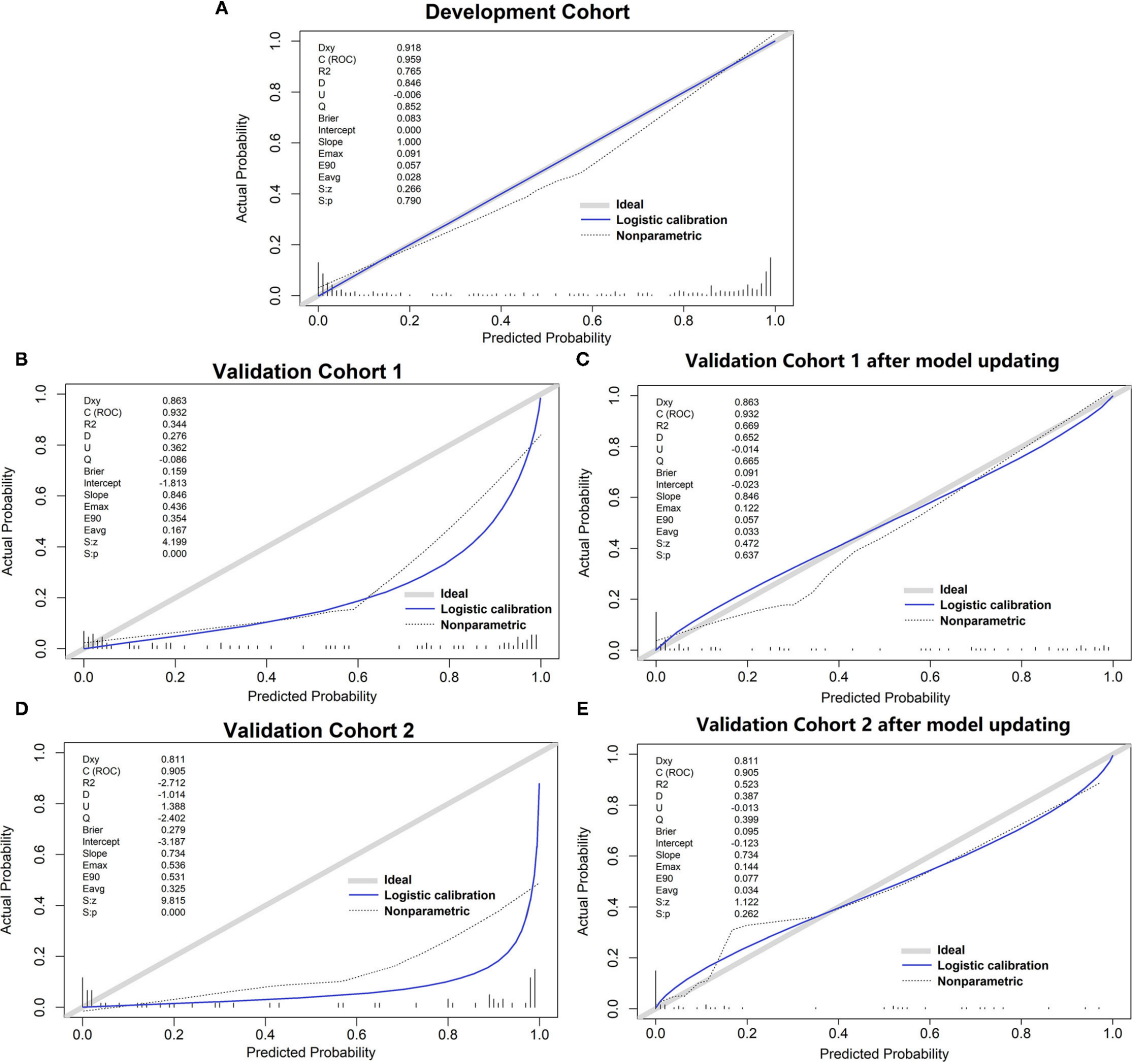
The calibration curve of different models. **(A)** development set; **(B)** validation cohort 1; **(C)** validation cohort 1 after model updating; **(D)** validation cohort 2; **(E)** validation cohort 2 after model updating.

The variables PT, age, TBil, and ALT were used to construct the logistic regression model called the PATA model. The prediction risk probability of ACLF can be calculated by the following model: linear predictor = 0.341 + 3.111^*^PT + 0.595^*^age + 0.626^*^TBil + (-0.295) ^*^ALT. Predicted risk probability = 1 / (1 + e ^∧^ linear predictor). The cut-off value for the high-risk and low-risk groups was 0.614 based on a balance of sensitivity and specificity (Table 2). The AUC of the model on the development set was 0.959 (0.941, 0.977). For two external validation cohorts, the AUC achieved 0.932 (0.876–0.987) and 0.905 (0.826–0.984) (Table 2 and Figure 2), which means that the PATA model has a high predictive effect for liver failure. The calibration curves have good linearity with the brier scores of 0.083, 0.159, and 0.279, respectively (Figure 3). The calibration of the model was assessed *via* the Hosmer–Lemeshow goodness-of-fit test (*P* = 0.147). We also performed a model update on the external validation set using a closed likelihood ratio test in Supplementary Methods and Supplementary Figure 4. The related results after updating were shown in Supplementary Tables 4, 5, Supplementary Figure 5, and Figures 3C,E.

**Table 2:** Performance of prediction model.

**Variable**	**Development**	**Validation**	**Validation**
	**cohort**	**cohort 1**	**cohort 2**
AUC (95% CI)	0.959 (0.941, 0.977)	0.932 (0.876, 0.987)	0.905 (0.826, 0.984)
Cutoff	0.614^*^	0.614	0.614
Sensitivity	0.894	0.909	0.923
Specificity	0.896	0.762	0.596
Accuracy	0.895	0.813	0.662
Positive predictive value	0.913	0.667	0.364
Negative predictive value	0.873	0.941	0.969

* *We chose cutoff based on a balance of sensitivity and specificity*.

### Nomogram and Online Tools

**Figure 4:**
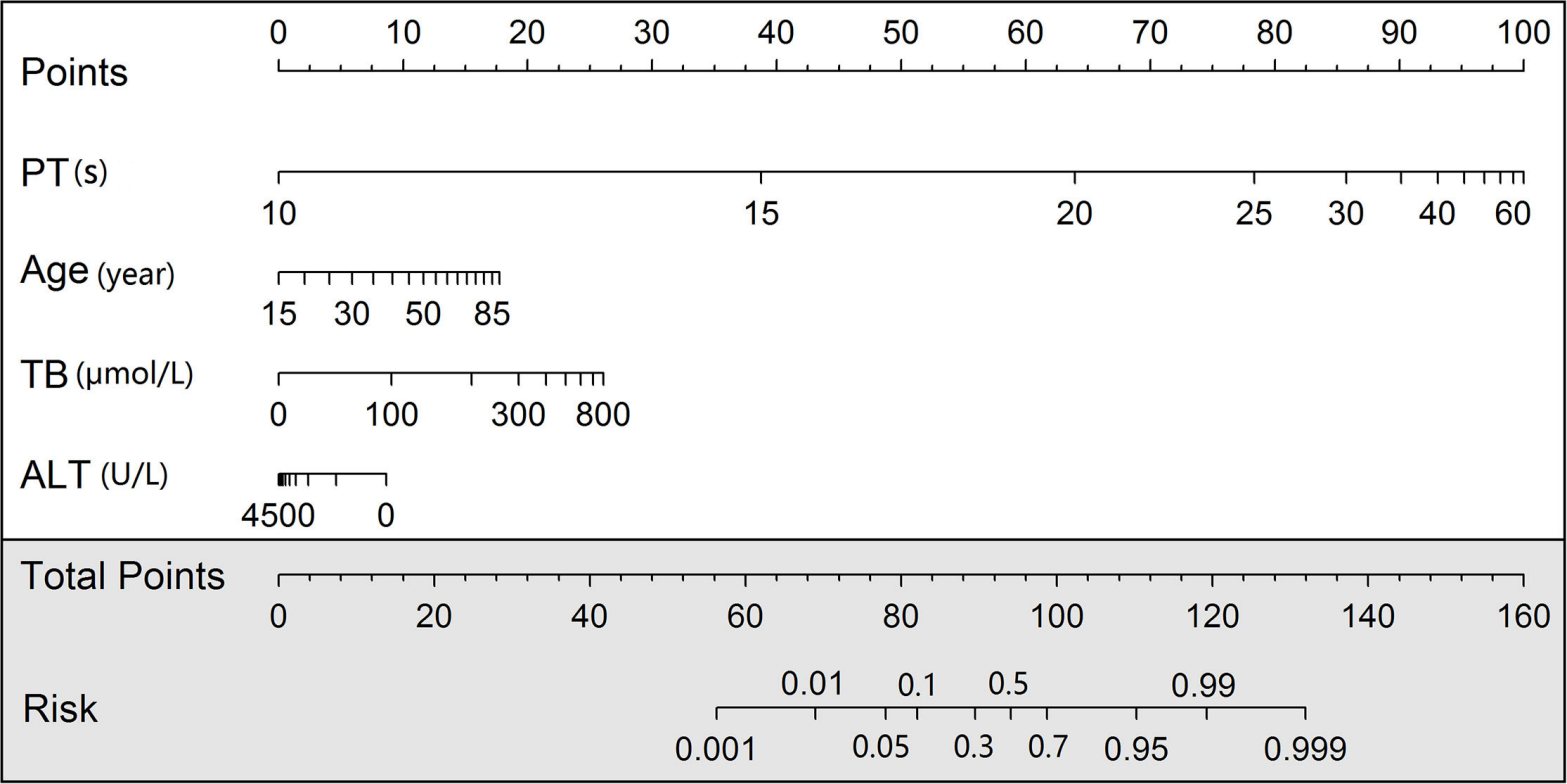
Nomogram for development set.prothrombin time (PT), Tbil, alanine aminotransferase (ALT).

We developed a nomogram scoring system and also a web page calculator to help physicians with quantitative scoring (Figure 4). The web interface created for clinicians allows the visualization of key information for risk prediction on cloud sever. This online application can be accessed by phone or computer but requires an internet connection for both private and public use (https://mia9510.shinyapps.io/MIA_LF/).

### Predictive Power for 90 Days Mortality Compared With MELD Score and CTP Score

**Figure 5:**
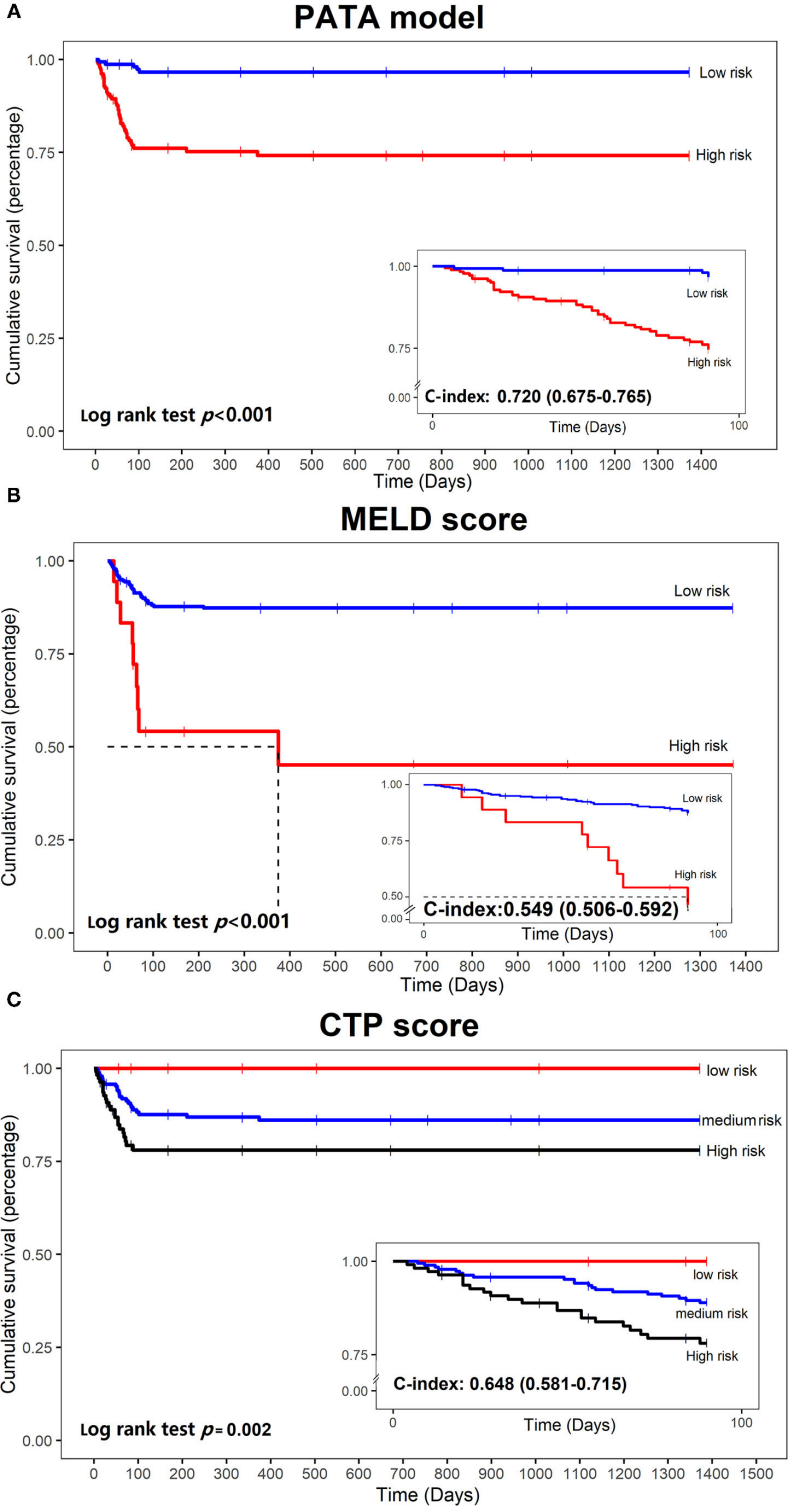
Kaplan–Meier curves of mortality curves according to the different risks of liver failure. **(A)** PATA model; **(B)** model for end-stage liver disease (MELD) score; **(C)** Child–Turcotte–Pugh (CTP) scoring system.

To explore the prognostic differences in the model risk stratified population, we compare our mortality prediction to the MELD score and CTP score in the development set. Patients were divided into a low-risk group (MELD score ≤ 30) and a high-risk group (MELD score > 30) for further analysis. Accordingly, patients were also divided into three groups based on CTPstage as follows: low-risk group (CTP-A), medium-risk group (CTP-B), and high-risk group (CTP-C). The stratification into different risk subgroups allowed significant distinction between Kaplan–Meier curves for survival outcomes (log *p* < 0.05). As shown in Figure 5, the PATA model outperformed the other models in 90 days of prognostic stratification for patients.The C-index was 0.720 (0.675–0.765) for our PATA model, 0.549 (0.506–0.592) for MELD score, and 0.648 (0.581–0.715) for CTP score.

## Discussion

Owing to the unpredictable outcome of rapidly progressing liver failure, early identification of ACLF is fundamental to implement appropriate preventive strategies in SAE patients with CHB. In recent years, several prognostic models have been developed for risk stratification of liver failure, but no predictive model has been widely accepted. In the current study of 503 patients with CHB with SAE from three medical centers, by using the RFE algorithm, we developed and validated a novel risk prediction model for ACLF using PT, age, TBil, and ALT. The predictive model demonstrated reasonably good discrimination and calibration. The AUC values of our PATA model were 95.9, 93.2, and 90.5% in the development cohort and two validation cohorts, respectively. The use of the PATA model may provide improved ability to early identify patients who are truly at increased risk of ACLF. We also compared the mortality predictive performance of the PATA model to that of the MELD score and CTP score. The results indicated that the PATA model showed better discrimination. The stratification of risk by the PATA model significantly improved on prediction and prognosis of ACLF for patients with CHB with SAE. It means that after screening high-risk groups by using the model, these patients not only have a higher incidence of liver failure but also a poor prognosis. Through this accurate prediction, the scoring system may be effective for guiding and optimizing therapeutic strategy. On the one hand, it can reduce the incidence of liver failure by early drug or artificial liver treatment, and on the other hand, it can make clear the prognosis of patients and prepare for liver transplantation.

Our PATA model with the best combination filtered by the RFE algorithm, including PT, age, TBil, and ALT. Older age has been identified as a risk factor in some studies (43, 44). So far, how the liver is affected by increasing age has not been fully elucidated. PT and TBil are commonly recognized as reliable markers of liver dysfunction (45–47). In this study and as well as previous studies (27, 29, 48), PT and TBil were significant independent risk factors for ACLF. Interestingly, low ALT level was an independent risk factor for progression to ACLF which was consistent with Yuan et al. (28) ALT level reflects the degree of hepatocyte necrosis resulting from acute injury and high levels of ALT persisted for several weeks after control of HBV and clearance of HBsAg from the circulation (49). Since a large number of liver cell necrosis occurs during liver failure, ALT in the blood decreases gradually, but bilirubin increases gradually, by the bilirubin-enzyme separation phenomenon (50), which is often the risk factor for the prognosis of patients with HBV-ACLF. Therefore, we assumed that early bilirubin-enzyme separation indicated a poor prognosis of SAE.

Our study has several strengths. First, we would like to emphasize that all variables were simple, readily available laboratory indices, and can be measured in the real-world clinical setting. When our model was applied to a new cohort, the cutoff recommended was the same as obtained from this study. The model can be updated as shown in Supplementary Methods (50–54) for improving transportability to other individuals if the new center has an expanded set of variables. Second, the prediction model does not require clinicians to perform complex calculations but simple, practical, and feasible calculations to be applied. Our online system supports mobile access, allowing physicians to assess in real-time and assist in decision making based on the results of the assessment. The model enables clinicians to more easily engage with the patient with CHB in a discussion of risk and thus enhance risk communication. Having a substantially high risk of ACLF could serve as a trigger to initiate more frequent clinical visits and more aggressive treatment. Third, by having two external validation sets, a fixed model was generated in the development set and then validated in another two hospitals.

We also acknowledge limitations to this work. First, more than a dozen definitions have emerged to describe ACLF. Our model was based on APASL 2019 using a population that was all Asian. We have not yet validated the performance of the model from other populations. Second, since the diversity existed between the training cohort and two external validation cohorts in this study, the performance of the model may be affected, especially PPV. We found that the incidence of liver failure in the training set was significantly higher indicating that liver injury severity in our patients was more aggressive. The Third Affiliated Hospital of Sun Yat-sen University hospital is a tertiary fixed-point hospital for hepatitis, almost all severe patients with liver diseases in Guangdong Province will come to see a doctor, so selection bias might exist in the recruitment of participants. Third, the at-risk patient populations may differ at baseline and several months after follow-up. Present models were generated using baseline data. Whether the model is suitable to use after situations of patients are changed during follow-up is questionable and needs further perspective experiment validation.

In summary, using data derived from a multicenter cohort, we constructed a novel prediction model that uses simple, readily available variables to predict ACLF and patient survival in patients with CHB with SAE. This model will empower clinicians and patients with more accurate, patient-specific information regarding the risk of ACLF. Identification of high-risk individuals may facilitate appropriate preventative options to reduce the occurrence of ACLF. Future studies are needed to confirm the applicability of our model in the clinical setting and to determine the effect of our model.

## Data Availability Statement

Theoriginal contributions presented in the study are included in the article/Supplementary Material, further inquiries can be directed to the corresponding authors.

## Ethics Statement

Thestudies involving human participants were reviewed and approved by Ethics Committee of the Third Affiliated Hospital of Sun Yat-sen University [(2018)02-384-01]. The patients/participants provided their written informed consent to participate in this study.

## Author Contributions

MY and XiaL conceived the study, drafted the manuscript, and was involved in the interpretation of the data. YL and ZL performed data analysis and interpretation. YJ, XinL, XS, SZ, YW,and WX assisted with the provision of study materials or patients, collection, and assembly of data. YC and ZL involved in revising the manuscript critically for important intellectual content. All authors read and approved the final manuscript.

## Funding

Thiswork was supported by a grant for National Key Science Research and Development Program of China (2018YFC1315400); Key Science Research and Development Program of Guangdong province (2019B020228001); Guangdong Basic and Applied Basic Research Foundation (2019A1515110166); Science and Technology Program of Guangzhou (201804010474); the 5,010 Project of Clinical Research in Sun Yat-sen University (2016009); and Natural Science Foundation of Guangdong (2018A030310272).

## Conflict of Interest

The authors declare that the research was conducted in the absence of any commercial or financial relationships that could be construed as a potential conflict of interest.

## Publisher's Note

All claims expressed in this article are solely those of the authors and do not necessarily represent those of their affiliated organizations, or those of the publisher, the editors and the reviewers. Any product that may be evaluated in this article, or claim that may be made by its manufacturer, is not guaranteed or endorsed by the publisher.

## Abbreviations

HBV: hepatitis B virus
CHB: chronic hepatitis B
ACLF: acute-on-chronic liver failure
SAE: severe acute exacerbation
ALT: alanine aminotransferase
AST: aspartate transaminase
Tbil: total bilirubin
PT: prothrombin time
Fib: fibrinogen
HBeAg: hepatitis B e antigen
HBsAg: hepatitis B surface antigen
INR: international normalized ratio
CI: confidence interval
ULN: upper limit of normal.
