# Supplementary material for: Development and Validation of a Novel Risk Prediction Model Using Recursive Feature Elimination Algorithm for Acute-on-Chronic Liver Failure in Chronic Hepatitis B Patients With Severe Acute Exacerbation
Source: Front Med (Lausanne). 2021 Nov 1;8:748915. doi: 10.3389/fmed.2021.748915 (PMC8591055; doi:10.3389/fmed.2021.748915)
Supplement: Supplementary file 1 [file Data_Sheet_1.DOCX]

**Supplementary Materials Online Content**

**Supplementary Methods**

**Supplement Tables**

Table S1. Parameters of data pre-processing

Table S2. Univariate analysis of the risk of liver failure

Table S3. Consistency of RFE selection over resamples.

Table S4. Comparison results between different updated-models

Table S5. Parameters of updated-model selection

Table S6. Methods to update prediction models

**Supplement Figure**

Figure S1. (A) Missingness map for development set. (B) Missingness map for validation cohort 1. (C) Missingness map for validation cohort 2.

Figure S2. Bubble chart of correlation between variables

Figure S3. A plot of feature selection by recursive feature elimination (RFE). RFE algorithm selects the optimal combination of variables with the highest Area Under the receiver operating characteristic Curve (AUC).

Figure S4. Flow chart of calibration model selection for external validation cohorts.

Figure S5. The Area Under the receiver operating characteristic Curve (AUC) and Precision-Recall (PR) curves of different models after updating. The AUROC curves of (A) development set; (B) validation cohort 1; (C) validation cohort 2. The PR curve of (D) development set ; (E) validation cohort 1; (F) validation cohort 2.

**Supplementary methods**

*1 Recursive Feature Elimination (RFE)*

We used a recursive feature elimination (RFE) algorithm^51^, which is a backwards selection procedure, to determine if predictors would be advantageous. In searching for the optimal combination of variables using the RFE algorithm, we used a logistic regression model for modeling, and we performed 5 repeats of 10-fold cross validation to avoid overfitting. In this total of 50 cross-validations, we use a resampling method without replacement, where we randomly select 90% of the subset from the development set for training each time and measure the model performance on the remaining 10%. The RFE process starts by removing the least important metrics and then recreating the model and evaluating the performance, in a recursive loop. The absolute value of the standardized regression coefficient was used to determine the importance of each predictor. We also conducted a t-test of the model coefficients for each predictor 50 times with the original hypothesis of mean equal to 0 (**Table S4 in the supplementary**) to demonstrate that the coefficients of all four variables selected for our final model were statistically significant.

The parameters of the RFE algorithm are as follows:

| **Function** | **Parameters** |
| --- | --- |
| function 'rfeControl' | method = 'repeatedcv'; repeats = 5 |
| function 'rfe' | metric = "ROC"; method = "glm" |

*2 Model updating*

When clinic prediction models are fitted with logistic regression, researchers probably encounter a poorer performance in new individuals compared with that found in the development study^20, 52^. In order to improve the transportability or generalizability of the predictive performance of the model, we update, adjust and recalibrate the model in two external validation cohorts. Since model updating require collecting data prospectively, we suggest that hospitals can perform model updating when the amount of data meets the condition of EPV 10^53^. Otherwise, we suggest that they use the cutoff=0.614 obtained from the development set in this study.

2.1 model application and updating

In this study, we developed the PATA model with the cut-off value of 0.614 for the high-risk and low-risk groups. But when clinic prediction models are fitted with logistic regression, researchers probably encounter a poorer performance in new individuals compared with that found in the development study^52^. In order to improve the transportability or generalizability of the predictive performance of the model, model updating is recommended by the TRIPOD guidelines^20^. Since model updating require collecting data prospectively or retrospectively, we suggest that hospitals can perform model updating when the amount of data meets the condition of EPV 10^53^. Otherwise, we suggest that they use the cutoff of 0.614 in this study.

2.2 Model updating recommendations for two external validation hospitals

As we can see in the main text, the calibration curves for the two external validation hospitals of the PATA model showed a degradation in performance without updating. Considering that sufficient retrospective data were already available for the two external validation hospitals, we performed an updating of the PATA model. It can be seen that the updated model performs better by evaluating the brier scores and calibration curves of the updated model. Therefore, we recommend the use of the updated model for both external validation hospitals.

2.3 A closed likelihood ratio test method for model updating

Several methods for updating original prediction models have been proposed and evaluated in published papers^54^. In this study, we used a statistical method with a closed likelihood ratio test to select the most appropriate model for the target population, which classifies model updates into three types: updating the intercept, updating the intercept and slope, and re-estimation coefficients^52^. This method uses a series of likelihood ratio tests to compare different model update methods, and the final update strategy is selected by the p-value of the likelihood ratio test as follows and **Table S6** as well as **Figure S4**.

Step1: model4 vs model1, if p >0.05, choose model1, else continue;

Step2: model4 vs model2, if p >0.05, choose model2, else continue;

Step3: model4 vs model3, if p >0.05, choose model3, else model4, end.

*3 Brier score*

The brier score is used to measure the accuracy of probabilistic predictions. It is used for tasks in which the prediction must assign probabilities to a set of mutually exclusive discrete outcomes. The detailed formula is as follows:

$$BS = \frac{1}{N}\sum_{t=1}^{N} {(f_{t}-o_{t})}^{2}$$

In which, $f_{t}$ is the predicted probability, $o_{t}$ is the actual label, and *N* is the total number of samples.
