# Supplementary material for: Development and Validation of a Novel Risk Prediction Model Using Recursive Feature Elimination Algorithm for Acute-on-Chronic Liver Failure in Chronic Hepatitis B Patients With Severe Acute Exacerbation
Source: Front Med (Lausanne). 2021 Nov 1;8:748915. doi: 10.3389/fmed.2021.748915 (PMC8591055; doi:10.3389/fmed.2021.748915)
Supplement: Supplementary file 2 [file Data_Sheet_2.docx]

**Supplement Tables**

| **Table S1. Parameters of data pre-processing** | | | |  |  |  |
| --- | --- | --- | --- | --- | --- | --- |
| **Dataset** | **Items** | **PT** | **Age** | **TB** | **ALT** |  |
| **Development Cohort** | *γ* | -0.977 | -0.143 | 0.443 | 0.336 |  |
|  | Mean | 0.970 | 2.911 | 21.673 | 24.918 |  |
|  | SD | 0.013 | 0.144 | 8.266 | 9.205 |  |
| **Validation Cohort 1** | *γ* | -1.594 | 0.345 | -0.013 | 0.439 |  |
|  | Mean | 0.620 | 7.670 | 4.386 | 52.254 |  |
|  | SD | 0.003 | 1.048 | 0.804 | 13.172 |  |
| **Validation Cohort 2** | *γ* | 0.149 | 0.404 | 0.137 | 0.060 |  |
|  | Mean | 3.454 | 8.768 | 6.772 | 8.946 |  |
|  | SD | 0.278 | 1.696 | 1.522 | 0.795 |  |
| *γ*: YeoJohnson transformed coefficients gamma; SD: Standard Deviation | | | | | | |

| **Table S2. Univariate analysis of the risk of liver failure** | | |
| --- | --- | --- |
| **Characteristics** | **AUC** | ***p* value** |
| PT | 0.945 | <0.05 |
| TB | 0.843 | <0.05 |
| Fibrinogen | 0.735 | <0.05 |
| ALB | 0.728 | <0.05 |
| PLT | 0.716 | <0.05 |
| Age | 0.672 | <0.05 |
| HGB | 0.662 | <0.05 |
| WBC | 0.631 | <0.05 |
| HBV-DNA | 0.620 | <0.05 |
| HBeAg | 0.589 | <0.05 |
| ALT | 0.576 | <0.05 |
| HBsAg | 0.575 | <0.05 |
| BMI | 0.550 | <0.05 |
| AST | 0.487 | 0.793 |
| Gender | 0.461 | 1.000 |
| Cr | 0.425 | 1.000 |

| **Table S3. Consistency of RFE selection over resamples.** | | |
| --- | --- | --- |
| **Predictors** | **Number of Times Selected** | **In Final Model** |
| Age | 50 | Yes |
| PT | 50 | Yes |
| TB | 47 | Yes |
| ALT | 42 | Yes |
| WBC | 3 | No |
| HBsAg | 2 | No |
| HBV-DNA | 1 | No |
| ALB | 1 | No |
| HBeAg | 1 | No |
| BMI | 0 | No |
| HGB | 0 | No |
| PLT | 0 | No |

| **Table S4. Comparison results between different updated-models** | | | | | |  | | |  | |  |  |
| --- | --- | --- | --- | --- | --- | --- | --- | --- | --- | --- | --- | --- |
| **Dataset** | **Items** | | **Original model** | **Model with  updated intercept** | | **Logistic Recalibration** | | | **Model Revision** | |  |  |
| **Validation Cohort 1** | Intercept | | 0 | -1.77 | | -1.52 | | | - | |  |  |
|  | Shrinkage factor^*^ | | 1.00 | 1.00 | | 0.85 | | | - | |  |  |
|  | Residual deviance | | 85.29 | 59.99 | | 59.30 | | | 55.48 | |  |  |
|  | Df | | 96 | 95 | | 94 | | | 91 | |  |  |
|  | LRT Chisq p-value | | <0.001 | 0.27 | | - | | | - | |  |  |
|  | AUC (95% CI) | | 0.93  (0.87 to 0.98) | 0.93  (0.87 to 0.98) | | 0.93  (0.86 to 0.98) | | | 0.95  (0.89 to 0.99) | |  |  |
| **Validation Cohort 2** | Intercept | | 0 | -3.83 | | -2.94 | | | - | |  |  |
|  | Shrinkage factor^*^ | | 1.00 | 1.00 | | 0.73 | | | - | |  |  |
|  | Residual deviance | | 115.62 | 38.92 | | 37.75 | | | 33.32 | |  |  |
|  | Df | | 65 | 64 | | 63 | | | 60 | |  |  |
|  | LRT Chisq p-value | | <0.001 | 0.31 | | - | | | - | |  |  |
|  | AUC (95% CI) | | 0.90  (0.81 to 0.97) | 0.90  (0.80 to 0.97) | | 0.90  (0.82 to 0.97) | | | 0.92  (0.88 to 1) | |  |  |
| ^*^The regression coefficient on the linear predicted value of the original model | | | | | | | | |  | |  |  |
| **Table S5. Parameters of updated-model selection** | | | | |  | |  |  | |  | |  |
| **Dataset** | | **Model choose** | | | **Intercept** | | **PT** | **Age** | | **TB** | | **ALT** |
| **Development Cohort** | | **-** | | | 0.341 | | 3.111 | 0.595 | | 0.626 | | -0.295 |
| **Validation Cohort 1** | | Original model | | | 0.341 | | 3.111 | 0.595 | | 0.626 | | -0.295 |
|  | | Model with updated intercept^*^ | | | -1.774 | | 3.111 | 0.595 | | 0.626 | | -0.295 |
|  | | Logistic Recalibration | | | -1.524 | | 2.633 | 0.504 | | 0.529 | | -0.250 |
|  | | Model Revision | | | -1.694 | | 3.273 | 0.727 | | -0.128 | | -0.626 |
| **Validation Cohort 2** | | Original model | | | 0.341 | | 3.111 | 0.595 | | 0.626 | | -0.295 |
|  | | Model with updated intercept^*^ | | | -3.832 | | 3.111 | 0.595 | | 0.626 | | -0.295 |
|  | | Logistic Recalibration | | | -2.937 | | 2.284 | 0.437 | | 0.459 | | -0.217 |
|  | | Model Revision | | | -3.469 | | 1.989 | 1.165 | | 1.400 | | -0.274 |
| ^*^ Final models for extranal validation cohorts | | | | |  | |  |  | |  | |  |

**Table S6 Methods to update prediction models^#^**

| Model | Label | Parameter | Number of parameters |
| --- | --- | --- | --- |
| Model 1 | Original model | None | 0 |
| Model 2 | Recalibration in the large | Intercept | 1 |
| Model 3 | Recalibration | Intercept and slope | 2 |
| Model 4 | Model revision | Re-estimate coefficients | *p** |

**p* is equal to the number of regression coefficients (intercept not considered) in the original model.

^#^ This table is reported by Pavlou, M et al. ^53^
