# Supplementary figures and images for: Development and Validation of a Novel Risk Prediction Model Using Recursive Feature Elimination Algorithm for Acute-on-Chronic Liver Failure in Chronic Hepatitis B Patients With Severe Acute Exacerbation
Source: Front Med (Lausanne). 2021 Nov 1;8:748915. doi: 10.3389/fmed.2021.748915 (PMC8591055; doi:10.3389/fmed.2021.748915)

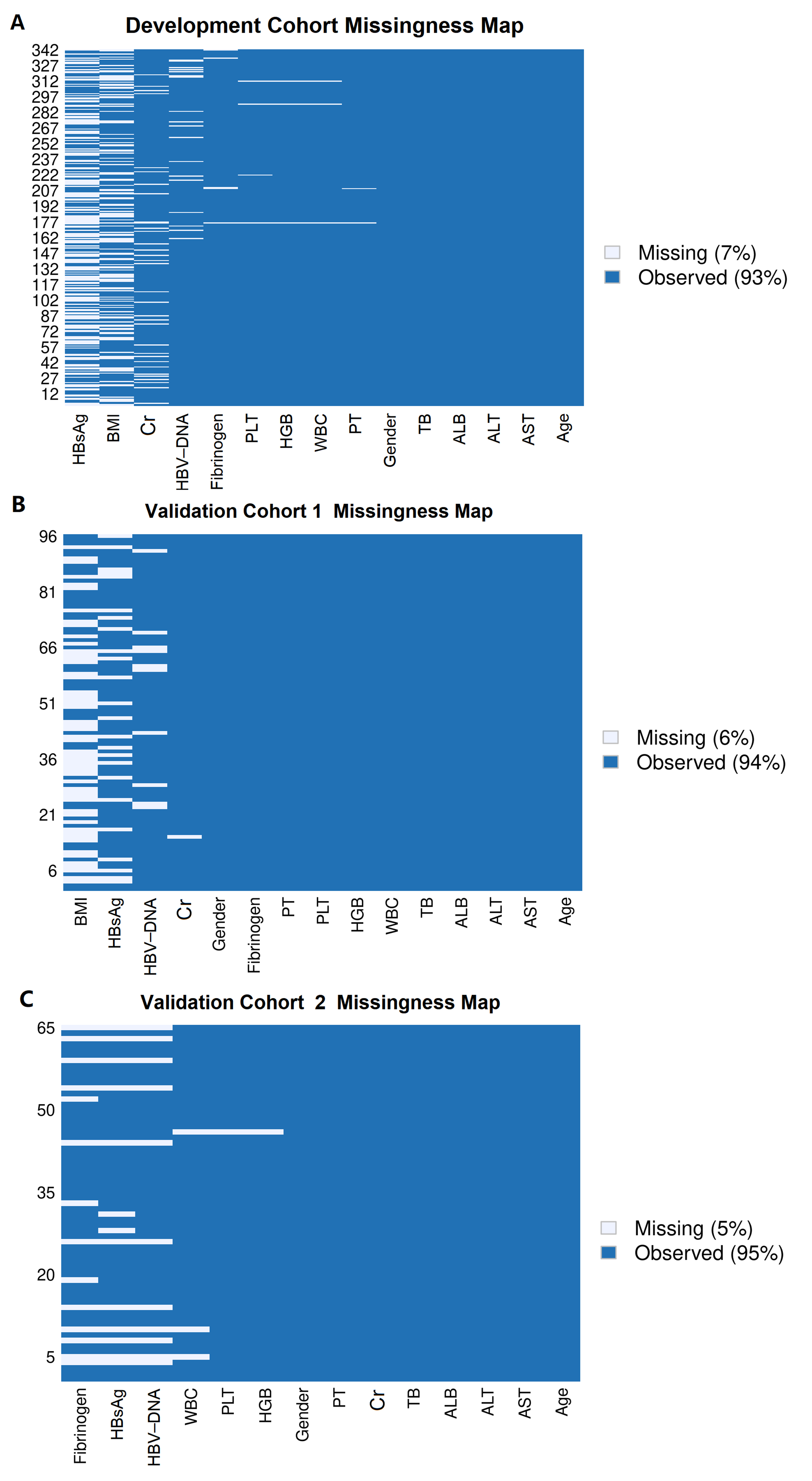

Supplement: Supplementary file 3 [file Image_1.tif]

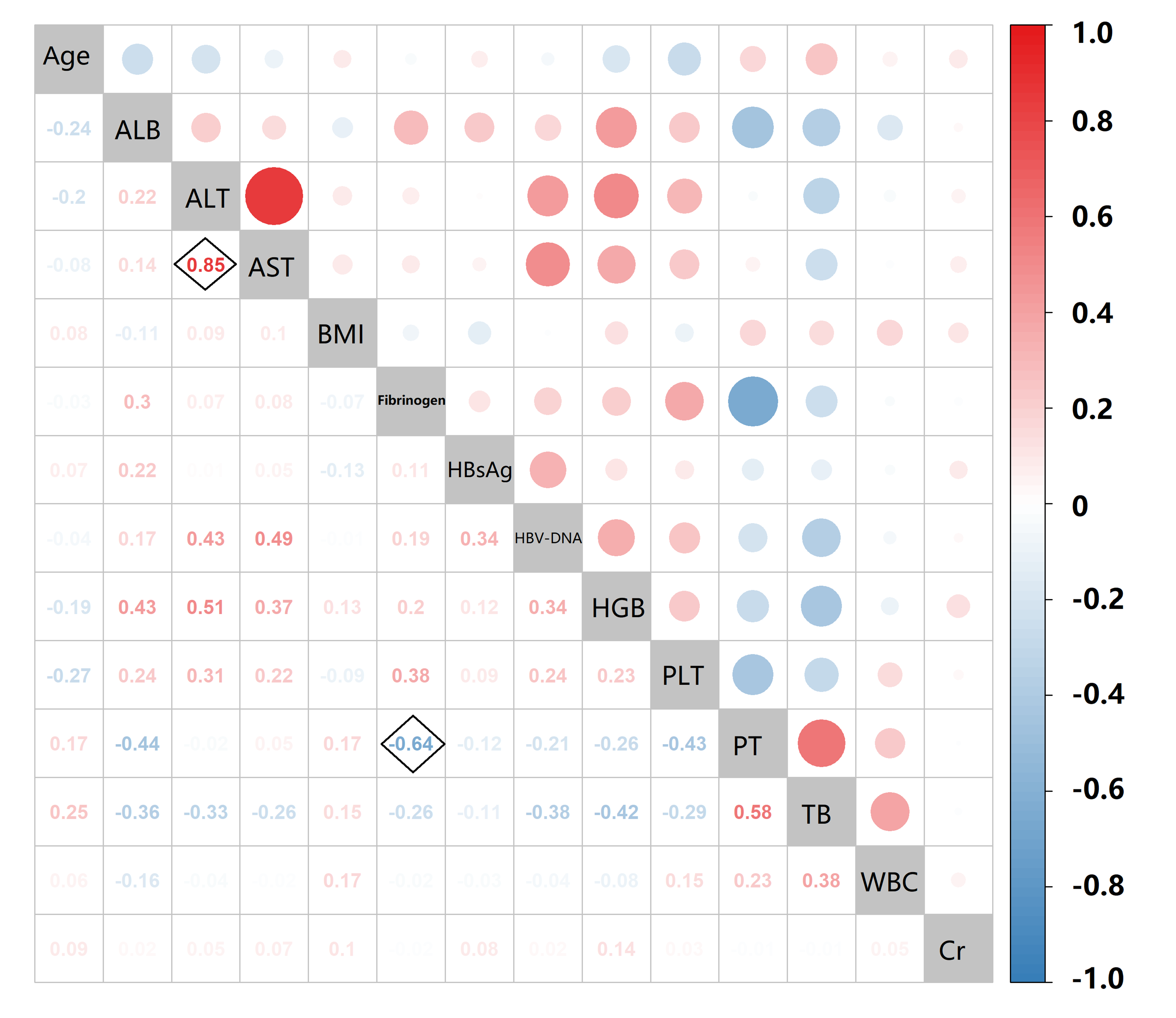

Supplement: Supplementary file 4 [file Image_2.tif]

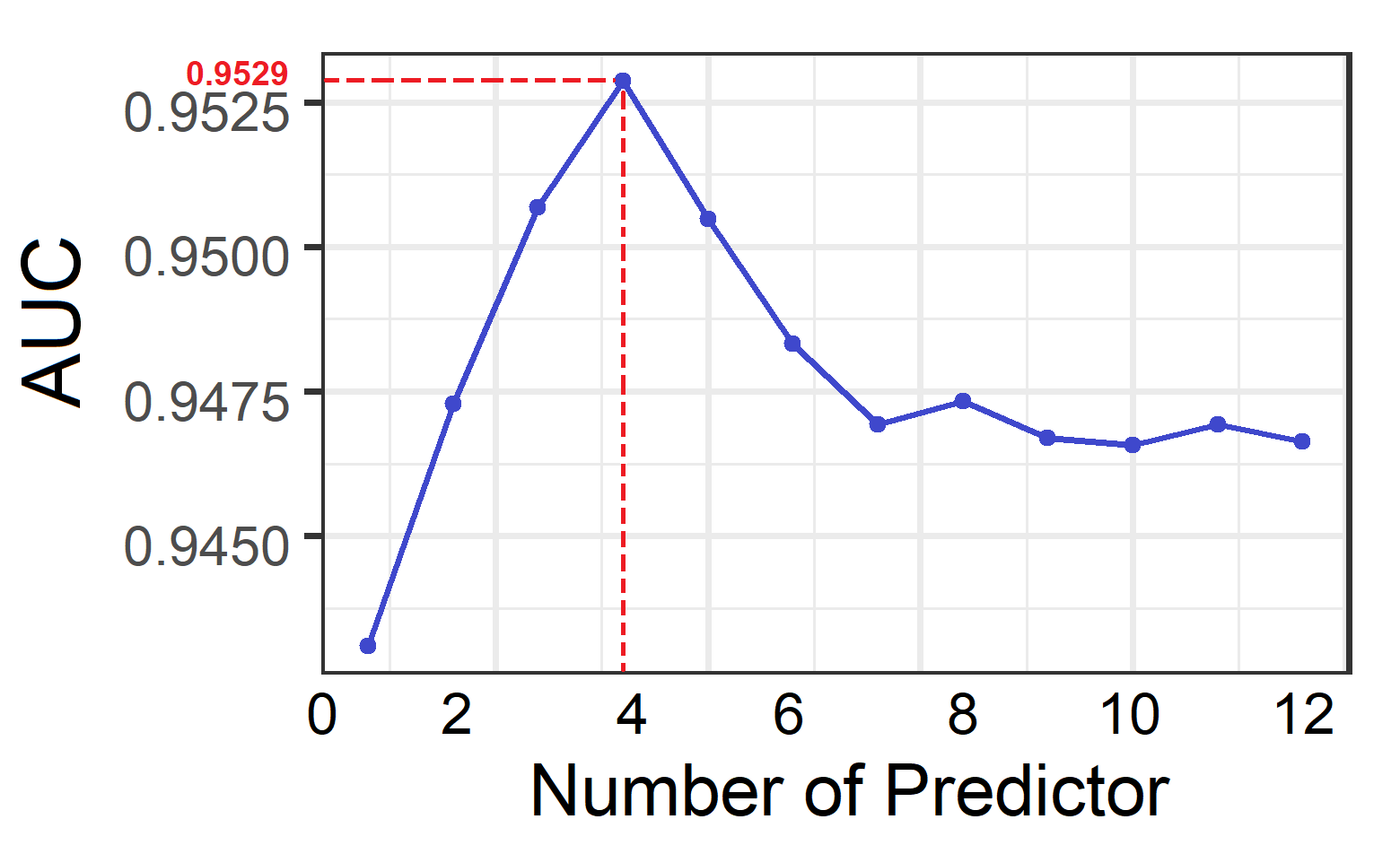

Supplement: Supplementary file 5 [file Image_3.tif]

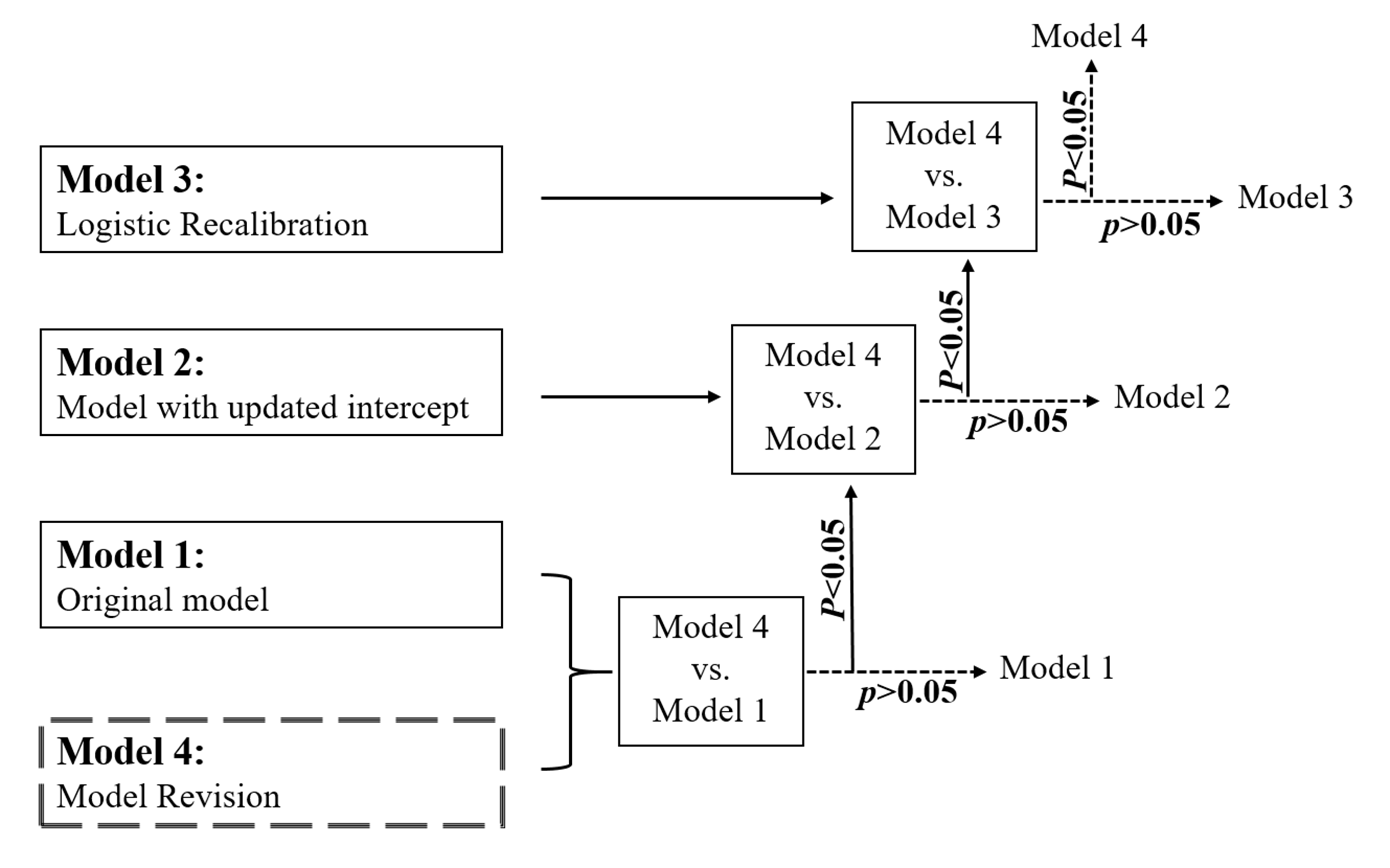

Supplement: Supplementary file 6 [file Image_4.tif]
